# Homology Modeling, Molecular Dynamics Simulation, and Prediction of Bovine TLR2 Heterodimerization

**DOI:** 10.3390/ijms25031496

**Published:** 2024-01-25

**Authors:** Alireza Mansouri, Mohamed Samy Yousef, Rasoul Kowsar, Akio Miyamoto

**Affiliations:** 1 Global AgroMedicine Research Center (GAMRC), Obihiro University of Agriculture and Veterinary Medicine, Obihiro 080-8555, Japan; alireza_mansouri89@yahoo.com (A.M.); elmaamly20102002@yahoo.com (M.S.Y.); 2 Department of Theriogenology, Faculty of Veterinary Medicine, Assiut University, Assiut 71515, Egypt; 3 Department of Animal Sciences, College of Agriculture, Isfahan University of Technology, Isfahan 84156-83111, Iran; rasoul_kowsarzar@yahoo.com

**Keywords:** bovine toll-like receptors, dimerization, homology modeling

## Abstract

Toll-like receptor 2 (TLR2) is a major membrane-bound receptor with ligand and species specificity that activates the host immune response. Heterodimerization of TLR2 with TLR1 (TLR2/1) or TLR6 (TLR2/6), triggered by ligand binding, is essential to initiating the signaling pathway. Bovine TLR2 (bTLR2) heterodimerization has not been defined yet compared with human and mouse TLR2s (hTLR2 and mTLR2). The aim of the present study was to model bovine TLRs (TLRs 1, 2 and 6) and create the heterodimeric forms of the bovine TLR2 using molecular dynamics (MD) simulations. We compared the intermolecular interactions in bTLR2/1-PAM3 and bTLR2/6-PAM2 with the hTLR2 and mTLR2 complexes through docking simulations and subsequent MD analyses. The present computational findings showed that bTLR2 dimerization could have a biological function and activate the immune response, similar to hTLR2 and mTLR2. Agonists and antagonists that are designed for hTLR2 and mTLR2 can target bTLR2. However, the experimental approaches to comparing the functional immune response of TLR2 across species were missing in the present study. This computational study provides a structural analysis of the bTLR2 interaction with bTLR1 and bTLR6 in the presence of an agonist/antagonist and reveals the three-dimensional structure of bTLR2 dimerization. The present findings could guide future experimental studies targeting bTLR2 with different ligands and lipopeptides.

## 1. Introduction

Toll-like receptors (TLRs) are membrane-bound proteins expressed in different types of cells, where they are involved in recognizing pathogen-associated molecular patterns (PAMPs) and mediating immune responses [1,2]. TLRs consist of an extracellular domain (ECD) that recognizes PAMPs, a transmembrane domain, and an intracellular toll-interleukin-1 receptor (TIR) domain for signaling [3].

The activation of TLRs relies mainly on their dimerization, where they pair with another TLR (hetero- and homodimerization). The binding of specific ligands to the ECDs of TLRs (TLR1-6) leads to dimerization by creating stable protein–protein interactions between TLR chains [4]. Those interactions are generated by hydrogenic bonding, non-covalent contacts, electrostatic forces, or hydrophobic effects between defined charged residues on the proteins [5]. TLR2 dimerization with TLR1 or TLR6 is required to initiate and mediate intracellular signaling during different innate immune responses in mammals. Quite recently, we reported the selective activation of the TLR2/1 heterodimer but not the TLR2/6 heterodimer to mediate the sperm-induced inflammation in the bovine uterus [6]. However, bovine TLR2 (bTLR2) heterodimerization has not been defined yet, at the molecular level, compared with human and mouse TLR2s (hTLR2 and mTLR2).

All available TLR2 agonists, such as Pam3CSK4 (PAM3) for the TLR2/1 heterodimer and Pam2CSK4 (PAM2) for the TLR2/6 heterodimer, are primarily synthetic lipoproteins designed for targeting hTLRs and mTLRs but not bTLRs [1,7]. Nevertheless, these agonists are usually used in bovine experimental models to activate the TLR2 signaling pathway [6,8,9,10,11]. Of note, TLR2 seems to play a key role in regulating the different reproductive events in bovines under physiological conditions [12,13]. Therefore, a better understanding of bTLR2 signaling/dimerization is now necessary and may lead to the development of therapeutic strategies that control the inflammatory responses in reproductive phenomena and improve fertility. Bovine immune cells showed lower responsiveness to TLR2 ligands (i.e., PAM3) compared to human cells [8]. This difference was due to the differences in ECDs between hTLR2 and bTLR2, while the TIR domains were similar across the species [8]. Therefore, the current study aimed to model the three-dimensional (3D) structure of the ECD of bovine TLRs (TLR1, 2, and 6) and design the dimeric form of TLR2 for comprehensive computational testing. This was achieved through modeled protein structure prediction methods, molecular dynamics (MD) simulations, and structural alignment techniques.

## 2. Results

### 2.1. Homology Modeling and Validation of Bovine TLRs

**Figure 1:**
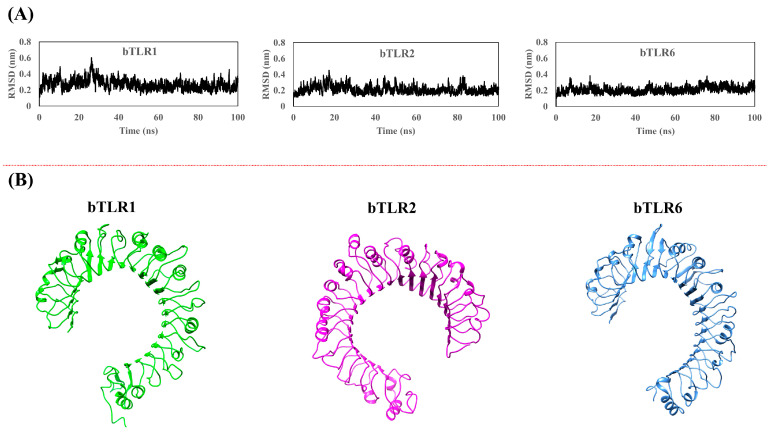
(**A**) RMSD plot after applying 100 ns MD simulation, confirming equilibration and structural optimization. (**B**) Optimized structures of bovine TLR1, TLR2, and TLR6 obtained through homology modeling followed by 100 ns MD simulation.

In the present study, we applied the homology modeling method to construct a 3D structure of bTLR2, bTLR1, and bTLR6. The available ECD structures of these TLRs, provided by the PDB, were used as templates along with the bovine constructed models. The initial models were subjected to MD simulation to define the stability of the models. Figure 1A illustrates the root-mean-square deviation (RMSD) of bTLRs during the MD simulation. The plot shows that all of the TLR models reached an equilibrium state after 30 ns of simulation and kept their stability up to the end of the dynamics (100 ns) (Figure 1A). The final snapshot obtained from the MD simulation revealed the typical horseshoe-shaped structures of the ECDs for bTLRs with an average RMSD of 2 Å (Figure 1B).

**Figure 2:**
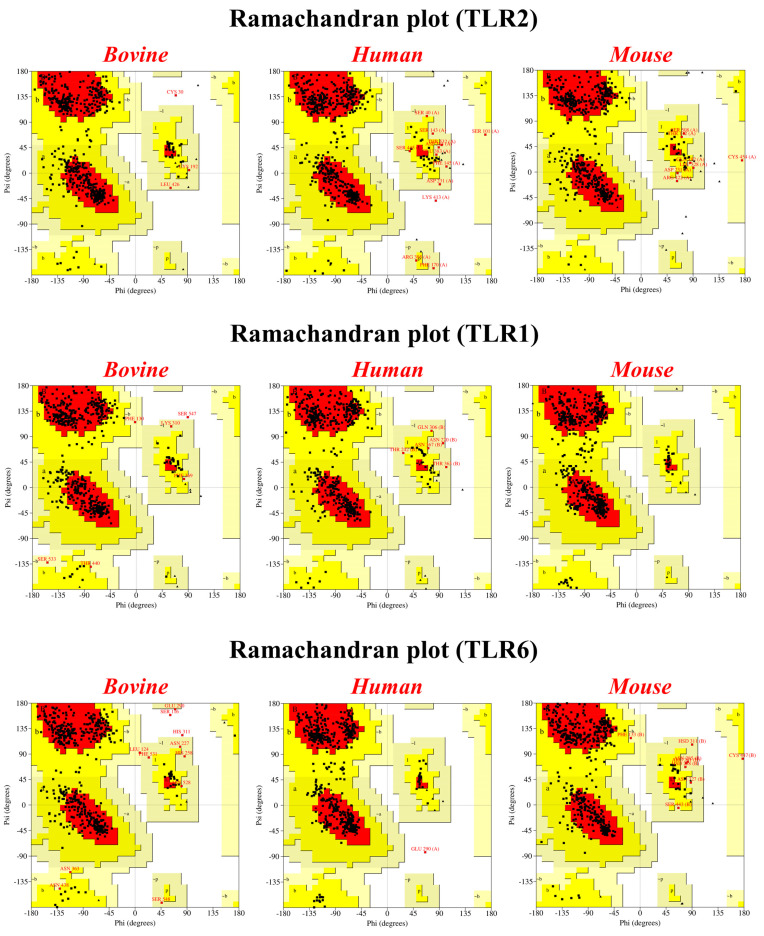
Ramachandran plot depicting the structural integrity of bovine, human, and mouse TLRs. The majority of residues fall within the allowed regions. Red (A, B, L), yellow (a, b, l, p) and pale yellow (~a, ~b, ~l, ~p) indicate the most favored, allowed and generously allowed regions, respectively. White color represents the disallowed regions.

The processed bTLR structures were validated for stereochemical parameters through the Ramachandran plot. The Ramachandran plot recorded that 100% of all residues in the allowed region for mTLR2, hTLR1, and mTLR6 originated from X-ray crystallography; it was 99.8% for hTLR2. Importantly, the Ramachandran plot showed the high accuracy of the bTLR structures that were obtained from homology modeling. It illustrated that more than 99% of residues in all the constructed models lay in the allowed region (Figure 2 and Supplementary Table S1). For bTLR2 and bTLR1, only one residue falls in the disallowed region (Cys30 and Ser547, respectively). Additionally, two residues are detected in the disallowed region for bTLR6 (Ser116 and His311) (Figure 2 and Supplementary Table S1). We also examined the Ramachandran plot for the modeled generated structures of hTLR6 and mTLR1. Since there are no crystal structures available for these TLRs, we utilized the structures obtained from the published UniProt database to assess them. These structures were predicted using the AlphaFold server. The results showed that all residues of mTLR1 (Q9EPQ1) fall within the allowed regions, while for hTLR6 (Q9Y2C9), there is one residue (Glu290) located in the disallowed region.

In addition, the overall quality factors were computed for TLRs in all species using the ERRAT program. This program assesses the quality of a protein’s 3D structure by analyzing the interaction of non-bonded atoms in the amino acids. A score exceeding 50 is generally indicative of a high-quality structure [14]. The ERRAT plots show an overall quality factor above 80% in all the TLRs of the studied species, signifying high-quality structures in human and mouse crystal structures, as well as in the modeled designed structures for bovines. For instance, the scores are 85.1 and 87.1 for bTLR1 and hTLR1, respectively. Regarding TLR2, the scores are 85.8, 86.6, and 87.7 for bTLR2, hTLR2, and mTLR2, respectively. Finally, for TLR6, the scores are 87.7 and 80.8 for bTLR6 and mTLR6, respectively. These results clearly demonstrate the highly accurate 3D structure generated for bovine TLRs (Supplementary Materials).

### 2.2. Generating Dimer Form of Bovine TLRs through Structural Alignment

**Figure 3:**
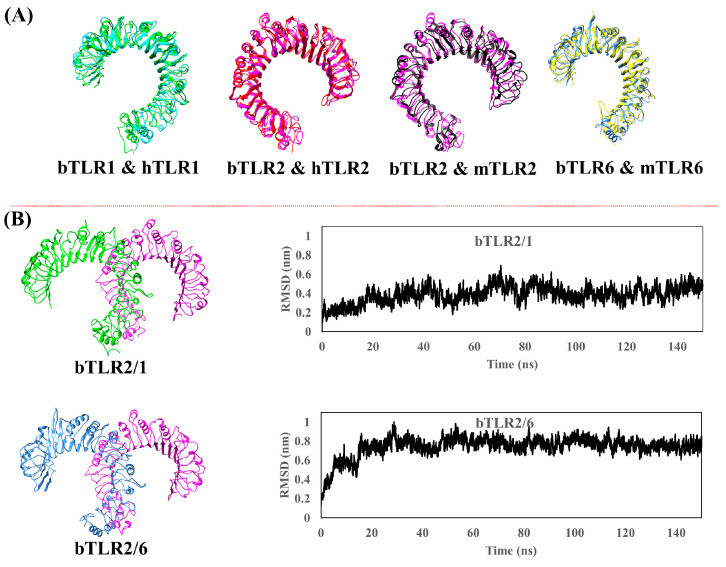
(**A**) Structural alignment of optimized bovine TLRs with their experimentally crystallized human and mouse counterparts. (**B**) Optimized bovine TLR2/1 and TLR2/6 heterodimers after applying 150 ns MD simulation. RMSD plots confirm the equilibration and the structural optimization.

Since the bovine TLRs have not been experimentally obtained, the crystal structures for the human (hTLR2/1) and mouse (mTLR2/6) TLR2 dimeric forms were utilized to align and recognize the new coordinates of bTLRs (Figure 3A) [1,2]. The new coordinates for bovine TLRs were acquired, isolated, and then merged to generate the heterodimeric form of bTLR2 (bTLR2/1 and bTLR2/6), followed by a 150 ns MD simulation (Figure 3B). The bTLR2/1 dimeric proteins displayed stability immediately upon the beginning of the simulation, with RMSD values between 0.2 and 0.4 nm (Figure 3B). In contrast, for TLR2/6, there was an increasing trend in the RMSD up to 20 ns (rising from 0.2 nm to 0.8 nm), followed by oscillations between 0.6 nm and 0.8 nm with an amplitude of 0.2 nm (Figure 3B). Upon validating the accuracy of the MD simulation, the trajectory was further utilized for analysis.

Additionally, we investigated the structural differences with the template modeling score (TM-score) using the TM-align tool [15]. Two protein structures with TM-scores > 0.5 seem to have a perfect match, while those with scores > 0.8 certainly have the same folding [16]. Interestingly, the scores obtained between bTLR2-hTLR2, bTLR1-hTLR1, bTLR2-mTLR2, and bTLR6-mTLR6 were 0.9, 0.8, 0.8, and 0.8, respectively.

The initial analysis involved calculating the binding free energy using the molecular mechanics/Poisson–Boltzmann surface area (MM/PBSA) method between the two TLRs in their dimeric forms. To accomplish this, the binding energies were computed in three different systems (in the absence/presence of the agonist and using the initial structures obtained from HADDOCK). The results of the MM/PBSA method are presented in Table 1 and Supplementary Table S2. In the application of protein–protein docking in the Haddock 2.4 web server [17], the residues participating in hydrogen bonding in TLR interaction were determined through Ligplot analysis [18]. These residues were identified as active sites at the contact interface of TLRs (Supplementary Figure S1). The HADDOCK web server was used to obtain protein–protein complexes, enabling the examination and validation of residues within the contact site between the two TLRs in their dimeric forms. The best conformer (from HADDOCK) was then used as the initial structure for the MD simulation, subsequently calculating the binding free energies. For the initialization of the MD simulation, cluster 1 was selected as the best-docked complex based on the superior HADDOCK score, computed using the following formula: Score = 1.0 * Evdw + 0.2 * Eelec + 1.0 * Edesol + 0.1 * Eair. The optimal HADDOCK scores for both TLR2 dimers are provided in Supplementary Table S3. Both van der Waals and hydrogen-bonding interactions contribute to the interface of TLRs in their dimeric forms. However, the MM/PBSA results unequivocally indicate a lack of significant affinity between the two TLRs, suggesting that TLR2 dimers cannot stabilize under physiological conditions in bovine species. The interaction energies between TLR2 and TLR1 averaged 423.7 kcal/mol (Table 1). Similarly, the interaction energy between TLR2 and TLR6 was measured at 240 kcal/mol.

The residues involved at the interface of the TLR2 dimers are illustrated in Supplementary Figure S1. There are notable similarities and differences between the two TLR2 dimers. In both dimers, Asp565, Arg571, Ser566, and Arg447 of TLR2 play crucial roles, engaging in hydrogen bonds with corresponding residues in TLR1 (Ser507, Glu535, Asn508-Gln531, and Ser436, respectively) and TLR6 (Ser508, Asn509, Cys533, and Trp412, respectively). However, distinct differences arise. In the TLR2/1 heterodimer, Lys490, Asn397, Arg398, Ser445-Glu369, and Asn345 of TLR2 form hydrogen bonds with residues in TLR1 (Asp561, Gln387, Lys385, Arg460, and Lys389, respectively). On the other hand, in TLR2/6, Glu375 and Asn534 of TLR2 establish hydrogen bonds with Arg342-Asn509 and Arg463 of TLR6, respectively.

### 2.3. The Interaction of TLR2 Agonists (PAM3 and PAM2) with Bovine TLRs

**Figure 4:**
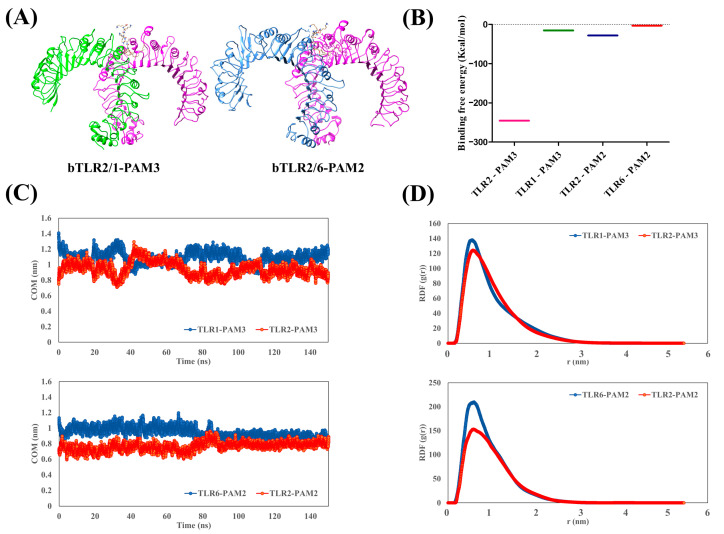
(**A**) Optimized structures of bovine TLR2/1-PAM3 and bTLR2/6-PAM2 after applying 150 ns MD simulation. (**B**) Binding free energy calculated for PAM3 and PAM2 with the main binding site of TLRs in the last 10 ns of MD simulation using MM/PBSA approach. (**C**) Center of mass (COM) analysis for PAM3 and PAM2 with the main binding sites of TLRs, demonstrating strong affinity. (**D**) Radial distribution function (RDF) of PAM3 and PAM2 with the main binding site of TLRs during MD simulation, confirming COM result with a peak at 0.5 nm.

To investigate the influence of agonists on bovine TLR2 dimerization, the agonists were initially docked into the main binding site of the bTLRs (Supplementary Figure S2). The docking details were adapted from agonist docking to human and mouse TLRs [1,2,6]. Following the docking studies, a 150 ns MD simulation was employed (Figure 4A). The RMSD plot of the protein backbone for two systems, bTLR2/1-PAM3 and bTLR2/6-PAM2, distinctly demonstrates the equilibration in both systems following a 150 ns MD simulation (Supplementary Figure S3). After the initial 70 ns, the RMSD fluctuated within the range of 0.6–0.8 nm and 0.2–0.3 nm for bTLR2/1-PAM3 and bTLR2/6-PAM2, respectively. The binding free energies (BFEs) obtained using MM/PBSA to predict the agonist’s binding in the bTLRs are presented in Table 1 and Figure 4B. The MM/PBSA results underscored that the interaction of the agonists with TLR2 is stronger than that found in TLR1 and TLR6. For instance, the BFE for TLR2-PAM3 was −245.5 Kcal/mol, whereas for TLR1-PAM3, a BFE of −15.4 Kcal/mol was observed. Regarding TLR2-PAM2, the BFE was −28.4 Kcal/mol, while for TLR6-PAM2, the BFE was −3.2 Kcal/mol. Additionally, the computed BFE was −245.5 kcal/mol for bTLR2/1-PAM3, and −36.1 kcal/mol for bTLR2/6-PAM2 (Table 1).

Moreover, the center of mass (COM) analysis demonstrated the stable interaction between the agonists and the main binding sites of the TLRs (Figure 4C). The amplitude of COM remained consistently below 1.5 nm, with minor fluctuations over the 150 ns MD simulation. Specifically, in the case of TLR2/1-PAM3, the distance between PAM3 and the main binding site of TLR1 initially measured 1.4 nm, fluctuating to approximately 1.2 nm during the simulation. A similar pattern emerged for PAM3 and the main binding site of TLR2, beginning at 0.8 nm and oscillating around 1 nm. For TLR2/6-PAM2, the distance between PAM2 and the main binding site of TLR6 fluctuated around 1 nm, while for TLR2, it was approximately 0.8 nm. These data collectively affirm the substantial stability of PAMs in proximity to the main binding sites of the TLRs.

In addition, the radial distribution function (RDF) was employed to explore the spatial distribution of PAMs around the binding site of the TLRs (Figure 4D). Peaks in the RDF curve signify areas of elevated PAM density in close proximity to the main binding site of the TLRs. Notably, a distinct peak emerged at approximately 0.8 nm, signifying the closest approach between the PAMs and the main binding site of the TLRs. These findings unequivocally demonstrate a robust affinity between the PAMs and the main binding site of the TLRs throughout the MD simulation.

The MD simulation time was extended to 300 ns to confirm the stability of the TLRs in the presence of an agonist. The COM distance between the agonist and the TLRs is illustrated in Supplementary Figure S4. The analysis of the COM data is indicative of the strong and stable interaction between the agonist and the TLRs over a 300 ns simulation.

**Figure 5:**
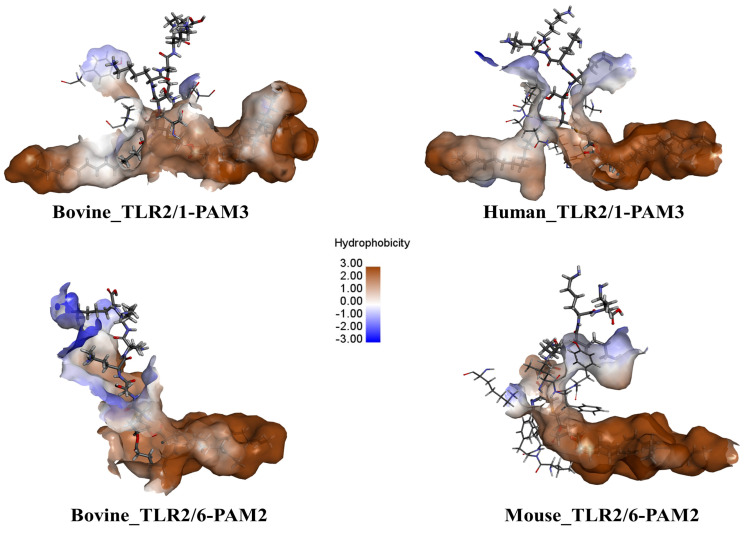
Residue types in TLR dimers interacting with PAMs in bovine, human, and mouse models. Brown denotes hydrophobic residues, while blue indicates polar or electrostatic residues. Acylated chains of PAMs engage in hydrophobic interactions, while the peptide section engages in electrostatic, H bonds, or polar interactions.

Figure 5 illustrates the hydrophobic interactions between the PAMs and the TLRs in the three different species. Due to the presence of hydrophobic residues within the central channel of the TLRs, hydrophobic interactions occur between the acylated chains of the PAMs and the main binding site of the TLRs. However, the peptide chain of the PAMs can engage in various types of interactions, including electrostatic, hydrogen bonding, and polar interactions, primarily with the polar residues in the binding sites of the TLRs. While the residues in the main binding site of the TLRs across all three species share a hydrophobic character, there are some differences observed. For instance, the hydrophobicity of hTLR2 is higher in the human TLR2/1 heterodimer than in its bovine counterpart. In addition, the hydrophilic part is larger in bTLR2/6 compared to mTLR2/6. The residues participating in H bonds and those involved in hydrophobic contacts are shown in Supplementary Figure S5, according to the Ligplot analysis. The types and number of residues involved in the interaction between the TLR2 heterodimers and the PAMs are summarized in Supplementary Table S4. The high number of polar residues in the bovine heterodimers can explain the presence of hydrophilic parts in these dimers when compared to humans and mice (Figure 5).

Additionally, a protein sequence alignment was conducted, including TLRs from three species: humans, bovines, and mice. The findings of this alignment are presented in Supplementary Figure S6. The analysis revealed variations in pocket site residues among these species. Specifically, in humans, Thr335, Pro306, and Leu266 were identified as occupying the pocket sites. In mice, these residues underwent mutations to Leu335, Leu306, and Phe266. In the case of bovine TLR2 (bTLR2), the critical residues were determined to be Thr335, Leu306, and Phe266. This comparative analysis sheds light on the species-specific variations in TLRs and their corresponding pocket site residues.

### 2.4. Interaction between TLR2/1 Antagonist (CU-CPT22) and TLRs of Humans, Mice, and Bovines

**Figure 6:**
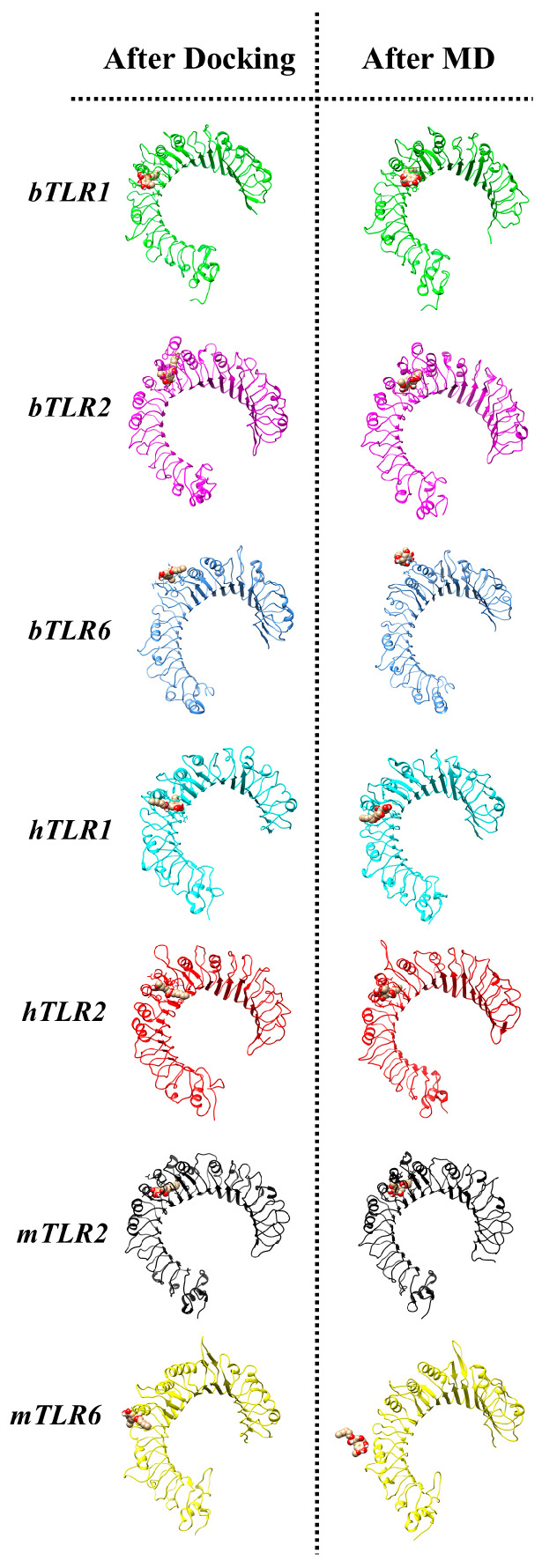
Initial (after docking) and final (after MD simulation) structures of the main antagonist (CU-CPT22) and TLRs in monomeric forms after 150 ns MD simulation for all three species. The antagonist can reside in the main binding sites of TLR2 and TLR1, but not TLR6.

**Figure 7:**
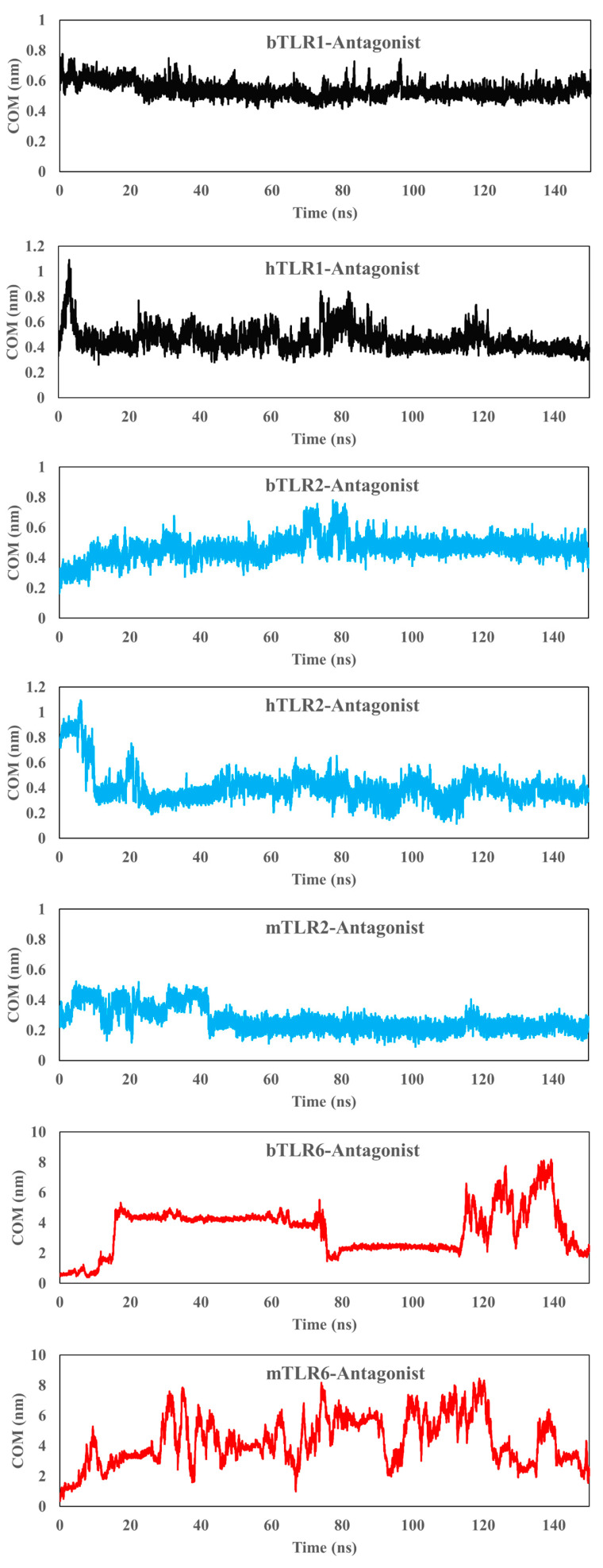
Center of mass (COM) plot for the main antagonist (CU-CPT22) and the main binding site of TLRs in monomeric forms. The data indicate a strong interaction between the antagonist and the main binding sites of TLR2 and TLR1. However, for TLR6, the antagonist cannot remain at the main binding site and occupies another pocket.

Initially, the antagonist was docked to the main binding site of the TLRs using dimeric and monomeric forms, and then a 150 ns simulation was conducted. The antagonist effectively binds to the main binding site of TLR1 and TLR2 in their monomeric forms and fails to bind to TLR6 across all three species (Figure 6). Furthermore, the COM analysis affirms a strong affinity between the antagonist and TLR1 and TLR2 (Figure 7). For instance, in the case of TLR1, the COM starts at approximately 0.5 nm (in the initial docking structure), and the antagonist maintains this position over the simulation time. Similarly, for TLR2 in all species, the variation in COM is minimal and reaches a stability state around 0.4 nm after approximately 40 ns. On the other hand, there is an absence of interaction between the antagonist and the main binding site of bovine and human TLR6, with a sharp increase in the COM plot after 20 ns.

To provide further clarity on the interaction between the antagonist and the bovine TLRs, we conducted RMSD calculations for both the bound and unbound states of the antagonist and the TLRs [19]. The results are illustrated in Supplementary Figure S7. The RMSD analysis indicated a lower deviation in the bound states compared to the unbound states, underscoring the interaction between the antagonist and TLR2 and TLR1. However, this difference was not significant for TLR6–antagonist complex structures, as expected. Additionally, we calculated the distance between an oxygen atom of the ligand and a carbon alpha of the main residue in the binding site. The distance analysis aligns with the COM analysis between the antagonist and the main binding site of the TLRs, highlighting a strong affinity between the antagonist and TLR2 and TLR1, but not with TLR6 (Supplementary Figure S8).

Moreover, the RDF analysis for the antagonist and the main binding site of the TLRs in their monomeric forms aligns with the COM results (Supplementary Figure S9). Regarding TLR1 and TLR2 in all species, the first peak occurs approximately at 0.5 nm, signifying a strong interaction between the antagonist and the main binding site of TLR1 and TLR2 across all species. Conversely, for TLR6, the initial peak occurs at approximately 2 and 2.5 ns (in bovines and mice, respectively), confirming the absence of interaction between CU-CPT22 and TLR6.

**Figure 8:**
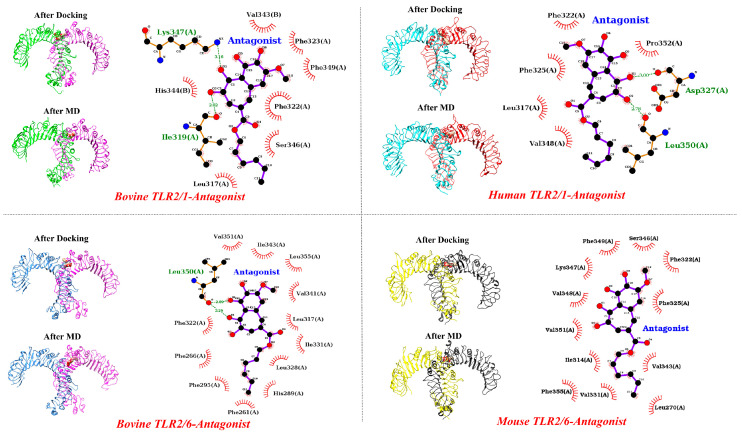
Initial (after docking) and final (after MD simulation) structures of the main antagonist (CU-CPT22) and TLRs in dimeric forms after 150 ns MD simulation. The antagonist can readily access the main binding site of TLR2, but not TLR1 and TLR6.

**Figure 9:**
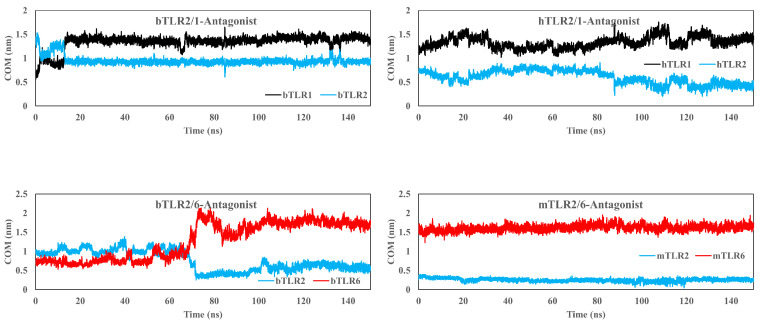
Center of mass (COM) plot for the main antagonist (CU-CPT22) and the main binding site of TLRs in dimeric forms. The data clearly indicate a strong interaction between the antagonist and the main binding sites of TLR2. However, for TLR1 and TLR6, the antagonist cannot remain at the main binding site and instead occupies the main binding site of TLR2.

Additionally, we docked CU-CPT22 to TLR2/1 and TLR2/6 dimers in all species and applied a 150 ns MD simulation (Figure 8). The antagonist engages with specific residues from both TLRs in their dimeric forms after docking simulation. However, following the 150 ns MD simulation, the antagonist migrates towards the main binding site of TLR2 (Figure 8). The Ligplot results indicate that, after the MD simulation, the residues of TLR2 are involved in the interaction with the antagonist in the TLR2/6 dimers in the bovine and mouse species, as well as in the TLR2/1 dimer in the human species. This implies that the antagonist effectively blocks the TLR2 main binding site to a greater extent than TLR1 or TLR6 in their dimeric forms. However, in the case of bovine TLR2/1, it appears that certain residues of TLR1 are also involved (His344 and Val343). For the TLR2/1 dimer and antagonist, the COM distance is smaller in TLR2 compared to TLR1. Interestingly, in bovine TLR2/1, the antagonist is positioned at the interface of the TLR dimer, and over time, the COM for TLR2 decreases. Meanwhile, the COM distance becomes notably higher for TLR6 after 70 ns compared with TLR2 (Figure 9). The reverse trend of the COM for TLR1 or TLR6 indicates the binding preference of the antagonist to the main binding site of TLR2 in its dimeric form. Moreover, the RDF analysis affirms a strong affinity between the antagonist and TLR2 relative to TLR1 and TLR6 across all species (Supplementary Figure S10).

### 2.5. Prediction of Glycosylation Sites in Bovine TLRs

**Figure 10:**
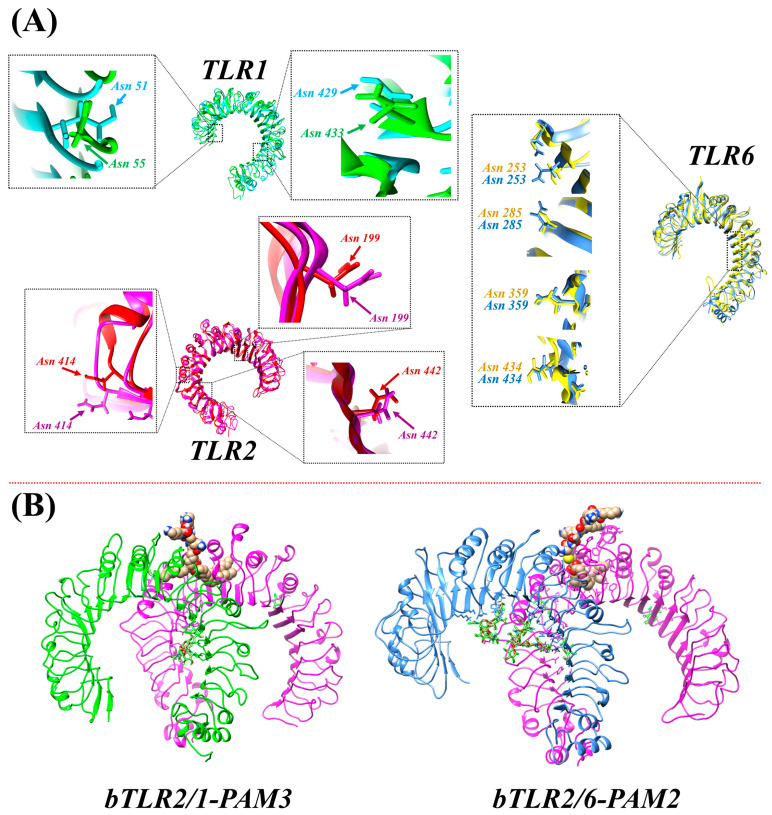
(**A**) Asn residues equivalent to those glycosylated in human and mouse TLRs, with corresponding Asn residues in bovine TLRs. (**B**) Optimized structures of bovine TLR2/1-PAM3 and TLR2/6-PAM2 after applying MD simulation. The TLR proteins were glycosylated at equivalent Asn residues based on human and mouse TLR glycosylated structures.

The glycosylation patterns of proteins play a pivotal role in their functions. However, these glycosylation patterns remain unknown in bTLRs due to the absence of crystal structures. To address this knowledge gap, we employed structural models of bTLRs from human and mouse TLRs. Initially, we conducted an in-depth analysis of glycosylated residues within the established crystal structures of human and mouse TLR2s (Supplementary Figure S11). Subsequently, we utilized a comprehensive structural alignment to clarify equivalent residues within the bTLR2 sequence that could potentially be glycosylated sites. Of note, the three Asn residues in human TLR2 have equivalent counterparts in bovine TLR2 (Asn199, Asn414, and Asn442) that are candidates for glycosylation (Figure 10A). Similarly, out of the four Asn residues in human TLR1, two residues (Asn51 and Asn429) find their equivalents in bovine TLR1 (Asn55 and Asn433). In the case of TLR6, out of the eight Asn residues in mouse TLR6, four have equivalent Asn residues in bovine TLR6 (Asn253, Asn285, Asn359, and Asn434). Following glycosylation of these equivalent Asn residues, the structural models of bovine TLR2 heterodimers were refined through a 150 ns MD simulation. The resulting structures in their dimeric forms are indicated in Figure 10B. The glycosylation sites apparently occur away from the main binding pocket in the 3D structure of all TLRs. Additionally, the optimized pdb structures are made available for researchers interested in further investigation.

## 3. Discussion

This study demonstrated the molecular basis of bovine TLR2/1 and TLR2/6 heterodimers and reported the response of these receptors to their agonists/antagonists compared to human and mouse structural models. Generally speaking, bovine TLR2 responded to lipopeptides in the same way as that of humans and mice. We showed the specific differences in the conserved residues in the heterodimerization interface across species.

TLR2 is associated with inflammation, apoptosis, and angiogenesis in various pathophysiological conditions in cattle (i.e., mastitis, endometritis, and enteritis) [20,21,22,23]. Quite recently, we reported the physiological function of TLR2 heterodimerization in the bovine uterus and the formation of the TLR2/1 dimer, but not TLR2/6, in response to sperm [6] via experimental and computational studies. In our previous investigations, we employed modeled modeling to construct the protein structure specific to bovine species [6]. Within this study, we identified the first predicted binding pockets in TLR1, TLR2, and TLR6 that were consistently located in the same positions across bovines, humans, and mice. Furthermore, it was revealed that the physicochemical characteristics of these pockets exhibited high similarities among all species. From these findings, we concluded that the process of dimerization is likely similar in humans, mice, and bovines [6]. However, the mechanism of interaction between agonists and antagonists with the main binding site of bovine TLRs remains unknown.

TLR2 monomers have previously been crystallized in complexes with their specific legend to mediate the heterodimerization process in humans and mice [1,2,24]. The binding of the tri-acylated lipopeptide, Pam3CSK4, to TLR ectodomains induced the formation of the TLR2/1 heterodimer, whereas the binding of the di-acylated lipopeptide, Pam2CSK4, activated the TLR2/6 heterodimer. Moreover, species-specific responses of TLR2 to lipoproteins have been reported [25] and explained by the structural variations observed in the TLR2 pocket [1]. For instance, Jin et al. [1] reported a significant difference in the sequences and structures of binding pockets in human and mouse TLR2s. In humans, Thr335, Pro306, and Leu266 occupy the pocket sites, whereas in mice, these residues are mutated to Leu335, Leu306, and Phe266. In the current study, these three crucial residues in bTLR2 were defined as Thr335, Leu306, and Phe266. Such differences can impact the sensitivity of the TLR2 response to their ligands across these three species. It is worth noting that we previously challenged the bovine endometrial epithelia with different TLR2 agonists/antagonists to induce/block a pro-inflammatory response [6,26]. However, bTLR2 heterodimers have no representative structures, which is a pre-requisite for understanding their function and mechanism of action.

In the present study, we employed homology modeling, structural alignment, and subsequent modeling of TLR2 dimeric forms for bovine species. Moreover, we conducted docking simulations of PAM3 and PAM2 into these binding pockets, revealing a notable affinity between the ligands and the main binding sites of TLRs.

This study revealed similarities among TLRs across the three species, emphasizing the presence of conserved regions and loops in the homologous TLR structures. Similar to human and mouse models, a lack of interaction between bovine TLRs in their dimeric forms was recorded in this study. These findings indicate that the process of TLR2 dimerization is not spontaneous in bovine physiology [6]. Previous studies have suggested that the dimeric form of TLR2 pre-exists, but the specific nature of this form remains unknown [1,2,27]. It has been proposed that bridging ligands, such as PAM3 and PAM2, can reconfigure the pre-existing forms and stabilize TLR2 dimeric structures in human and mouse models, as the pre-existing form exhibits weak inter-multimer binding [1,2]. In agreement with our results, the functional similarity of the TLR2 gene across vertebrates (including bovines) was established [28,29,30]. However, Turner et al. reported that, unlike mouse or human cells, the bovine endometrial cell responses to the diacylated lipopeptide involved TLR1, as well as TLR2 and TLR6. Such a difference may be due to using a different lipopeptide (FSL-1 instead of PAM2) [22].

In light of the unknown pre-formed TLR2 dimeric forms, our study assessed the antagonist’s effect on TLRs in both monomeric and dimeric states. Computational analyses unequivocally revealed that TLR2 antagonists effectively penetrate the main binding site of TLR2 across the human, mouse, and bovine species. Consequently, they obstruct and occupy this critical site and interfere with the dimerization process in the presence of agonists. Interestingly, the antagonist exhibits a blocking effect on TLR1 in both human and bovine models. However, our data did not indicate any interaction between the antagonist and the main binding site of TLR6. In dimeric forms, it becomes evident that antagonists exhibit a noticeable affinity for the main binding site of TLR2 across all species, surpassing their interaction with TLR1 and TLR6. This suggests that CU-CPT22 is able to antagonize TLR2 activation by both agonists (PAM3 and PAM2). Similar to our findings, Grabowski et al. demonstrated that CU-CPT22 robustly suppresses human TLR2/6 and mouse TLR2/1 via experimental and computational studies [31].

The prediction of protein–protein (or protein–ligand) interactions is greatly influenced by the accuracy of the structural models of the proteins. In this study, MD simulation was employed to refine the protein structures, and an overall quality factor was computed to assess the accuracy of these structures. The processed bTLR structure has been validated with a Ramachandaran plot having >99% residues in the allowed region and an overall quality factor exceeding 80% using the ERRAT tool. However, in real-world scenarios, proteins, as small biological structures, have abundant time to access their lowest free-energy ensembles. Hence, the computational approaches to predicting the proteins’ structure and their interactions are limited and must be experimentally tested.

Overall, this computational study provides structural analysis and predicts the protein–protein interactions of bTLR2 with bTLR1 and bTLR6 in the presence of an agonist/antagonist. It also reveals the three-dimensional structure of bTLR2 dimerization. The present findings could guide future experimental studies for a better understanding of the biological functions of bTLR2.

## 4. Materials and Methods

### 4.1. Phase I: Homology Modeling and Validation of Bovine TLRs

To obtain modeled generated structures of TLRs in bovine species, the protein sequences (amino acid sequences) were sourced from the UniProt database. Specifically, for TLR1, the code B5TYW4 (encompassing residues 25–544 (the extracellular domain)) was utilized. For TLR2, the code Q95LA9 (encompassing residues 27–579 (the extracellular domain)) was employed, and for TLR6, the code Q704V6 (encompassing residues 33–557 (the extracellular domain)) was selected. Subsequently, these sequences were input into SWISS-MODEL, an automated protein structure homology-modeling server renowned for its proficiency in this field [32]. SWISS-MODEL automatically identifies an appropriate template protein with a known 3D structure closely related to the target protein. It then aligns the target sequence with the template structure, utilizing this alignment to generate a 3D model of the target protein [32]. After obtaining the 3D structure, each structure was optimized using MD simulation.

Backbone conformation and stereochemical quality of our models were validated by analyzing Ramachandran plots derived from PROCHECK (version 3.5.) [33,34]. Moreover, the overall quality score was evaluated using the ERRAT program [35].

To identify the most accurate protein structure prediction, we utilized two well-known machine tools, SWISS-MODEL and AlphaFold. Both programs were employed to generate the structures of bovine TLRs. Subsequently, the generated structures were compared by assessing the overall quality score and analyzing the Ramachandran plot. The findings are presented in Supplementary Figure S12. The overall quality score unequivocally favored the structures obtained from SWISS-MODEL, surpassing those from AlphaFold. However, a structural alignment revealed strikingly similar folding patterns in TLRs between SWISS-MODEL and AlphaFold (Supplementary Figure S12). Consequently, despite the comparable structural alignment, our choice for computational analyses in this study leans towards SWISS-MODEL due to its higher overall quality score, acknowledging the potency of AlphaFold as well.

### 4.2. Phase II: Structural Alignment

The optimized structures obtained in Phase I were employed to generate the dimeric form of TLR2 in bovine species. To achieve this, each bovine TLR, along with its respective counterparts from humans and mice (forming the dimeric form), was subjected to structural alignment using TM-align [15]. Subsequently, the PDB file of TLR2 was merged with that of TLR1 and TLR6, resulting in the creation of the dimeric form of TLR2. Finally, each TLR2 heterodimer was further optimized through molecular dynamics (MD) simulations.

### 4.3. Phase III: Docking Simulation

The structures obtained in Phase II were utilized to conduct further investigations into the effects of TLR2 ligands (PAM3, PAM2, and CU-CPT22) on bovine TLR2 dimerization. For this study, docking parameters for bovine models were carefully selected, mirroring those used in human and mouse models (Supplementary Table S5). In order to warrant the accuracy of the selected docking parameters for bovine models, the agonist coordinates extracted from the human and mouse crystal structures were adopted to bovine TLR2 dimeric forms; subsequently, the docking structures were superimposed on the co-crystal structure coordinates. The superimposed structure with RMSD is depicted in Supplementary Figure S13. The minimal RMSDs (0.72 and 0.52 Å for docking PAM3 and PAM2, respectively) obtained affirm the accuracy of the docking parameters for this study in the bovine model. In detail, the structures of hTLR2/1, hTLR2, hTLR1, mTLR2/6, mTLR2, mTLR6, bTLR2/1, bTLR2/6, bTLR2, bTLR1, and bTLR6 were designated as receptors for TLR2 ligands. For both human and mouse TLRs, monomeric and dimeric models were acquired from the RCSB Protein Data Bank (PDB) using the crystal structure of human TLR2/1 (PDB: 2Z7X) and mouse TLR2/6 (PDB: 3A79). Specific grid box details were defined and applied to the main binding sites of TLRs in both monomeric and dimeric forms (Supplementary Table S4).

In the present study, we assessed the accuracy of the docking methods by utilizing PAMs, as they are well-established benchmarks for ligand-TLR2 interactions. The docking procedure was conducted using Autodock VINA and Autodock 4.2 with default parameters [36]. The obtained RMSD values of 0.51 Å (PAM3-TLR2/1) and 0.37 Å (PAM2-TLR2/6) using Autodock 4.2 confirmed the accuracy of our docking procedure. Notably, the outcomes from both docking programs were almost identical.

For the docking procedure, Autodock 4.2, coupled with a genetic algorithm (GA), was employed to investigate and predict the binding orientations of ligands to the main binding sites of the receptors [37]. The parameters included an initial population of random individuals with a size of 150 individuals, a maximum of 2.5 × 10^6^ energy evaluations, a maximum of 27,000 generations, a mutation rate of 0.02 (the probability of a gene undergoing a random change), and a crossover rate of 0.80 (the probability of two individuals undergoing crossover). Prior to docking, non-polar hydrogens and Gasteiger charges were added using AutoDockTools package version 1.5.6 [37]. Ligands were not permitted to freely rotate (non-rotatable bonds). Subsequently, 150 GA runs were performed for each docking run.

To validate our docking procedures, a binding energy of −28.36 and −4.75 kcal/mol and an RMSD of 0.5 and 0.38 Å were obtained for PAM3-TLR2/1 and PAM2-TLR2/6, respectively. The RMSDs of the two ligands were calculated through LigRMSD [38]. The superimposition of these conformers is illustrated in Supplementary Figure S14. The remarkably low RMSD value (below 2 Å, a widely accepted criterion) affirmed the appropriateness of our parameter selection for the docking procedure in this study [39].

After obtaining the structures from the docking simulation, each structure was optimized using MD simulation.

### 4.4. MD Simulation and MM/PBSA Calculations

MD simulations for free molecules consisted of four steps (minimization, NVT, NPT, and MD production) within a water box supplemented with Na^+^ and Cl^−^ ions to achieve an ionic strength of 140 mM [40]. The entire system was initially minimized using the steepest descent algorithm for 50,000 cycles without any positional restrictions. This was followed by an equilibration process consisting of a 100 ps NVT set of MD runs and, subsequently, a 100 ps NPT set with restrictions on TLRs and ligands at a 1000 kJ.mol^−1^.nm^−2^ harmonic force constant in the NPT phase. In the production step, 150 ns MD simulations were performed without any positional restraints for optimizing all molecules and TLRs/ligands. However, to ensure that the chosen simulation time was correct, the simulation time was extended for the two main systems (TLR2/1-PAM3 and TLR2/6-PAM2) in this study to 300 ns. The results are depicted in Supplementary Figure S4, and they strongly affirm the correctness of the simulation time selected in this study. The TIP3P water model was employed in this study to define the solvation box for the molecules, with a minimum distance of 1.5 nm between the solute and the box walls. The simulations were conducted at a temperature of 300 K, employing a time step of 2 fs and using the LINCS algorithm [41]. The periodic boundary condition (PBC) was considered in both the equilibration and production processes. The simulations were carried out using GROMACS 2020 with CHARMM 27 force field parameters [42,43]. The SwissParam website was utilized to obtain complete topologies of ligands based on CHARMM force field parameters [44]. Additionally, CHARMM-GUI was employed to model the N-glycan of TLRs (as illustrated in Supplementary Figure S11) [45]. In order to ensure the reproducibility of the MD simulation production data, two replicas for bovine TLRs and antagonist systems were conducted. The outcome of the MD simulation is depicted in Supplementary Figure S15. Consistent findings were observed across all replicates, affirming the strong reproducibility of the data.

In this study, the MM/PBSA method was chosen to compute the binding free energy between molecules using the MD production trajectories. The frames for generating the binding mode image were selected from the last 10 ns of the simulation (from 140 to 150 ns). This timeframe was chosen as the system had reached equilibrium, as confirmed by RMSD analysis. In the MM/PBSA approximation, snapshots were collected every 50 ps from trajectories after equilibration. Consequently, during 10 ns in total, 200 frames were used to calculate binding free energies. MM/PBSA calculates electrostatic, van der Waals, polar solvation, solvent accessible surface area (SASA), solvent accessible volume (SAV), and Weeks−Chandler−Andersen (WCA) energies. The binding free energy is the summation of these energies [46]. To validate the results derived from MD simulation and MM/PBSA, a comparative analysis was conducted by aligning our calculated binding free energy with established experimental data (Kd and KD) from previous studies [47]. Further, we investigated quantitative correlations between these three parameters (Kd, KD, and binding free energy) [47]. The compounds with experimental data (extracted from experimental research) were docked to the main binding site of human TLR2. The assessment of binding free energy involved MD simulation and the MM/PBSA method. Notably, the outcomes revealed a strong correlation coefficient (R^2^) between the binding free energy (determined via MM/PBSA) and the experimental KD (R^2^ = 0.77) and Kd (R^2^ = 0.60) data. The results are illustrated in Supplementary Figure S16.

## Figures and Tables

**Table 1:** Binding free energies (Kcal/mol) using MM/PBSA method for bTLR dimers in the last 10 ns of the MD simulation.

	TLR2–TLR1	TLR2–TLR6	TLR2/1–PAM3	TLR2/6–PAM2	TLR2–PAM3	TLR1–PAM3	TLR2–PAM2	TLR6–PAM2
bTLR2/1-PAM3 system	448.8	NA	−289.9	NA	−245.5	−15.4	NA	NA
bTLR2/6-PAM2 system	NA	339.6	NA	−36.1	NA	NA	−28.4	−3.2

NA: not applicable.

## Data Availability

Data available on request from the author.

## Supplementary Materials

The following supporting information can be downloaded at: https://www.mdpi.com/article/10.3390/ijms25031496/s1.
