# Supplementary material for: Homology Modeling, Molecular Dynamics Simulation, and Prediction of Bovine TLR2 Heterodimerization
Source: Int J Mol Sci. 2024 Jan 25;25(3):1496. doi: 10.3390/ijms25031496 (PMC10855669; doi:10.3390/ijms25031496)
Supplement: Supplementary file 1 [file ijms-25-01496-s001.zip › ijms-2798613-supplementary.pdf]

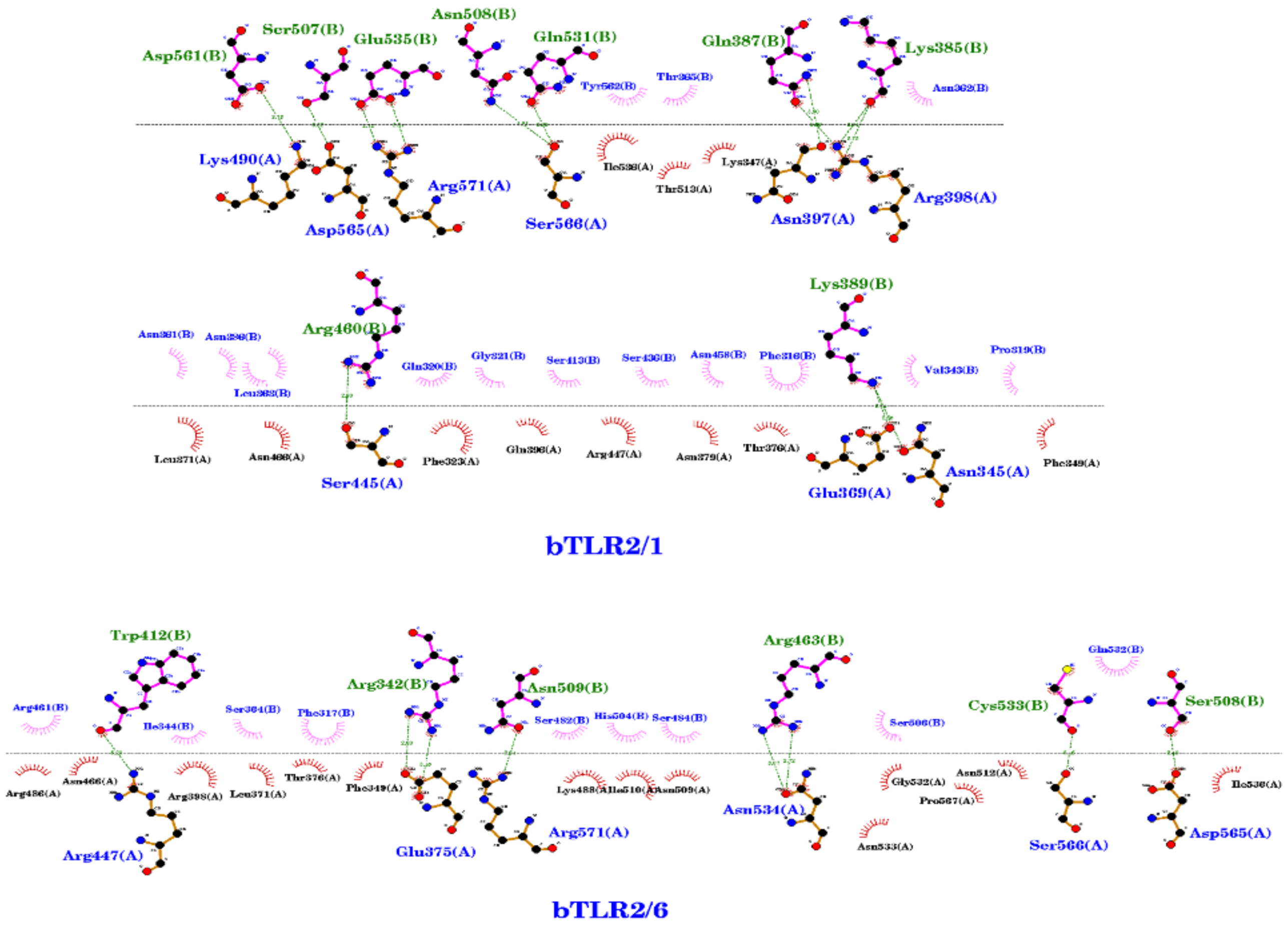

Supplementary Figure S1. Ligplot analysis of bovine TLR2 dimeric forms.

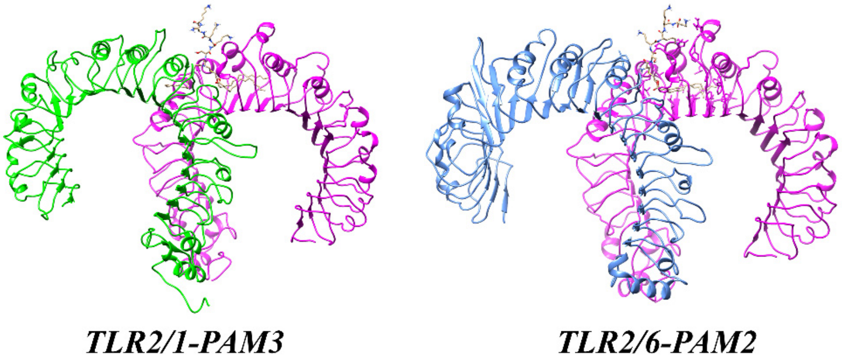

Supplementary Figure S2. The best docking mode of PAMs and TLR2 dimeric forms.

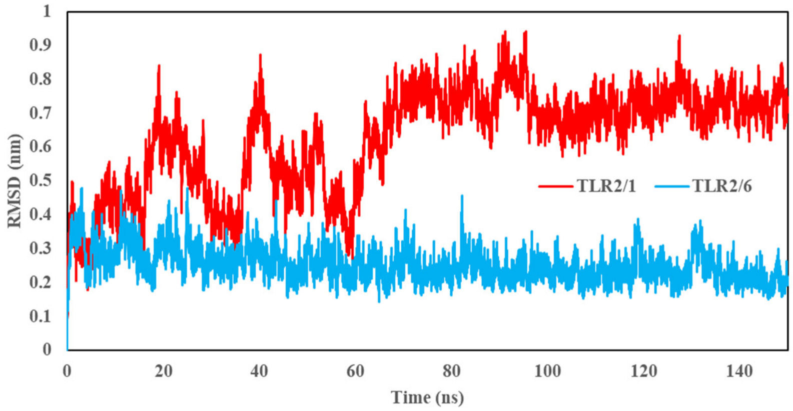

Supplementary Figure S3. RMSD plot for protein backbone for two systems (bTLR2/1-PAM3 and bTLR2/6-PAM2) during 150 ns MD simulation.

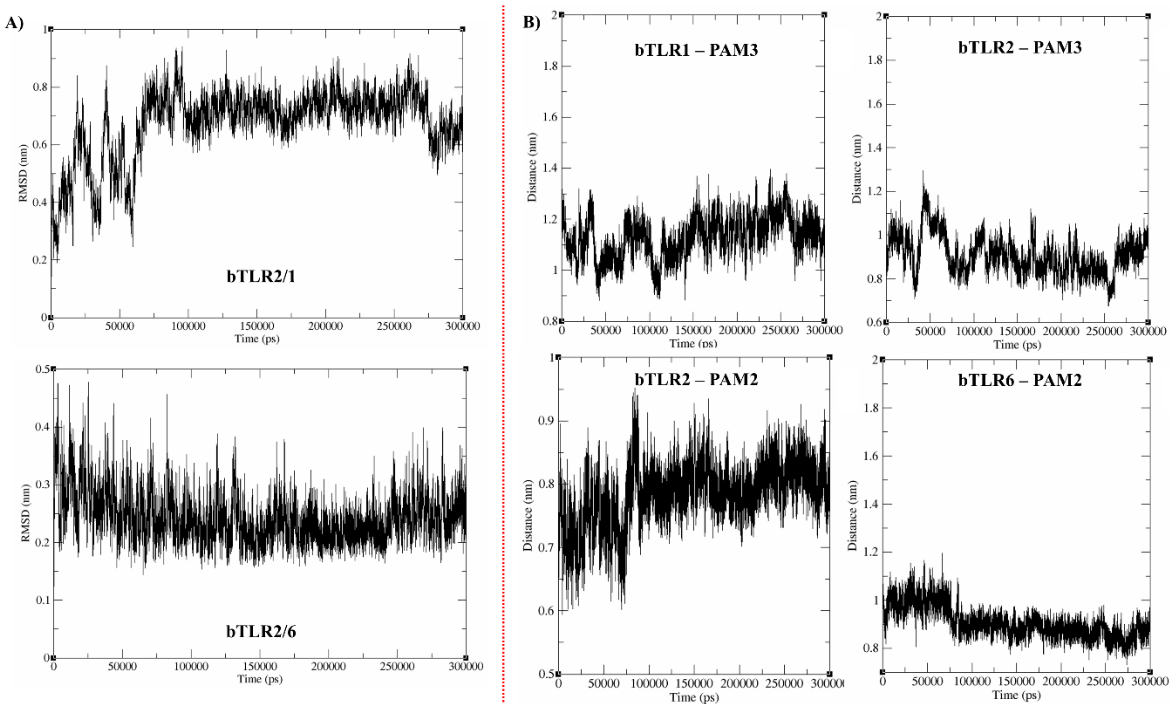

Supplementary Figure S4. Extension of MD simulation time to 300 ns for enhanced system stability over a longer duration. A) Analysis of the Root Mean Square Deviation (RMSD) of the protein backbone in the TLR2 heterodimer system. B) Analysis of the center of mass distance between TLRs and agonists.

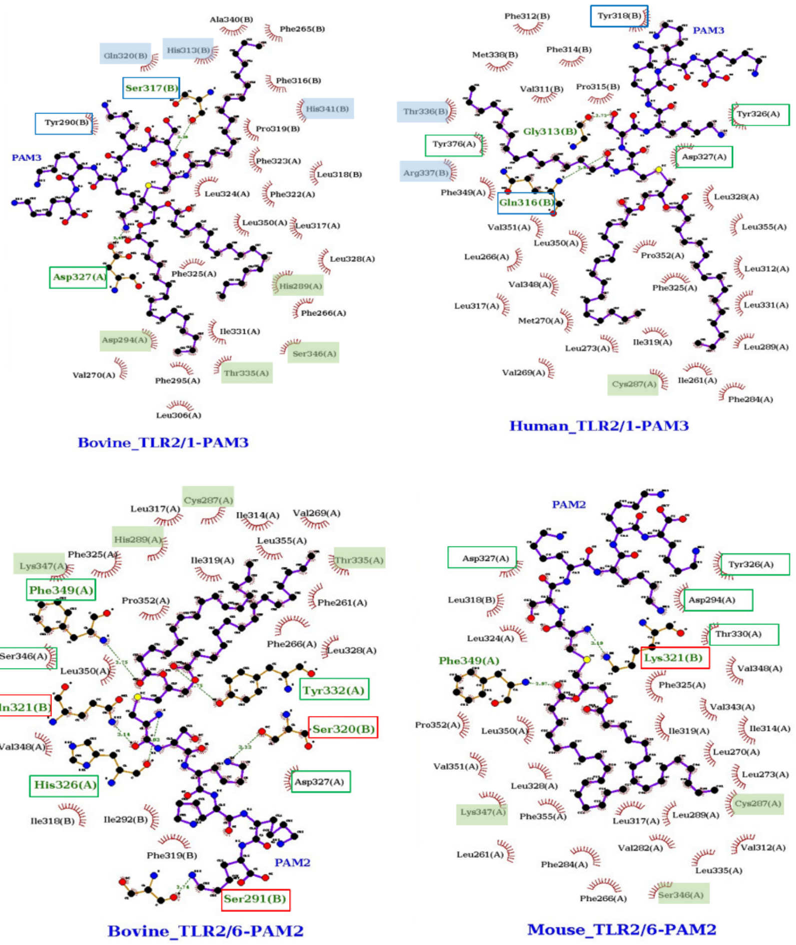

Supplementary Figure S5. Ligplot analysis illustrating residues involved in PAMs interactions with TLRs, with hydrophobic residues playing a dominant role. Green outlined boxes indicate polar residue of TLR2 involved in the interaction with peptide chain of PAMs. Blue outlined boxes indicate polar residue of TLR1 involved in the interaction with peptide chain of PAMs. Red outlined boxes indicate polar residue of TLR6 involved in the interaction with peptide chain of PAMs. Green semi-transparent boxes indicate polar residue of TLR2 involved in the interaction with acylated lipid chain of PAMs. Blue semi-transparent boxes indicate polar residue of TLR1 involved in the interaction with acylated lipid chain of PAMs.

TLR2
Human - Bovine

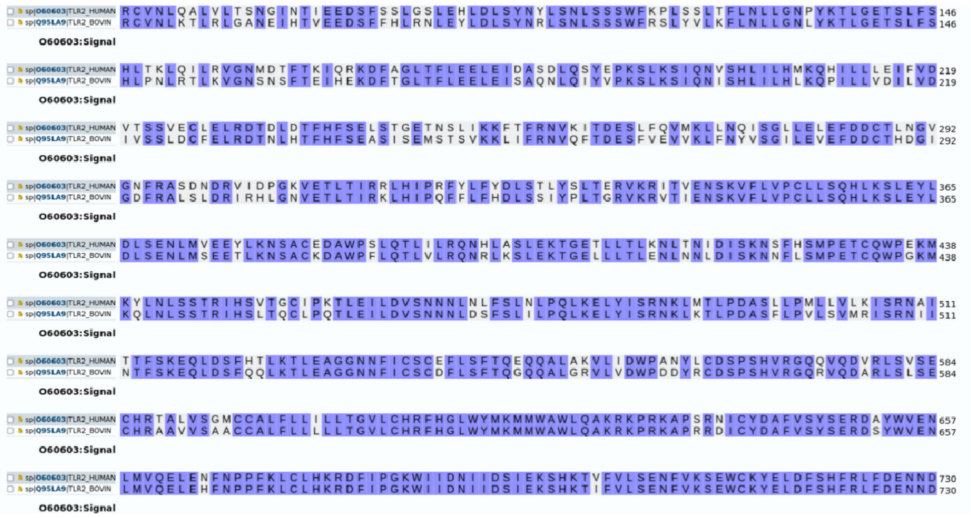

TLR2
Mouse - Bovine

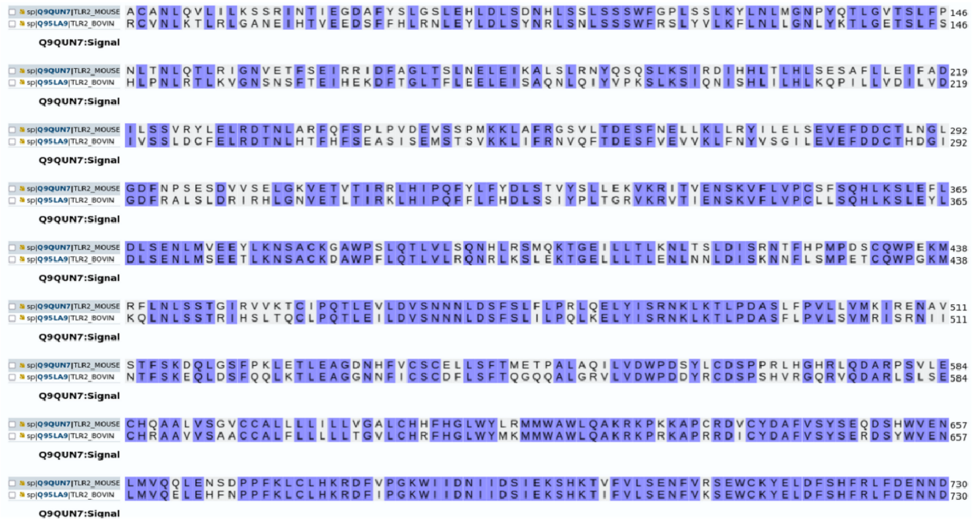

Supplementary Figure S6. Protein sequence alignment comparing human TLR2 with bovine TLR2, and mouse TLR2 with bovine TLR2.

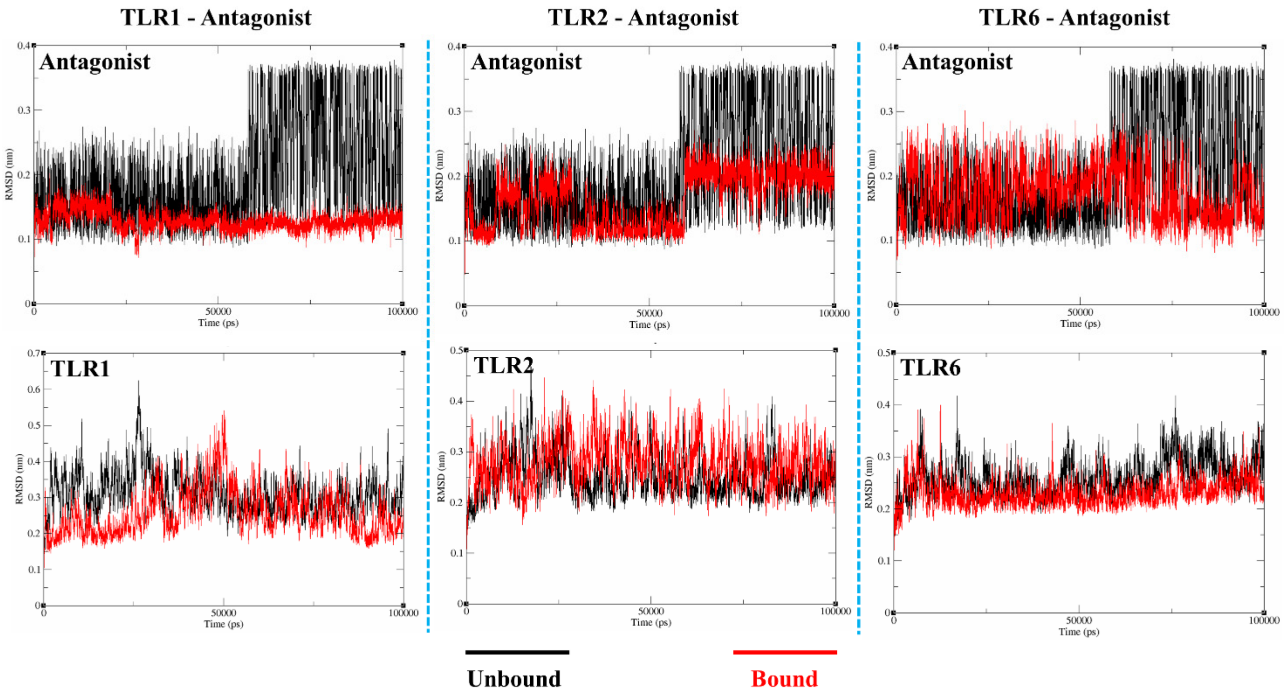

Supplementary Figure S7. RMSD of antagonist and bovine TLRs during 100 ns MD simulation. The RMSD analysis indicates a lower deviation in the bound states compared to the unbound states, underscoring the interaction between the antagonist and TLR2 and TLR1. However, this difference is not significant for antagonist-TLR6 complexes.

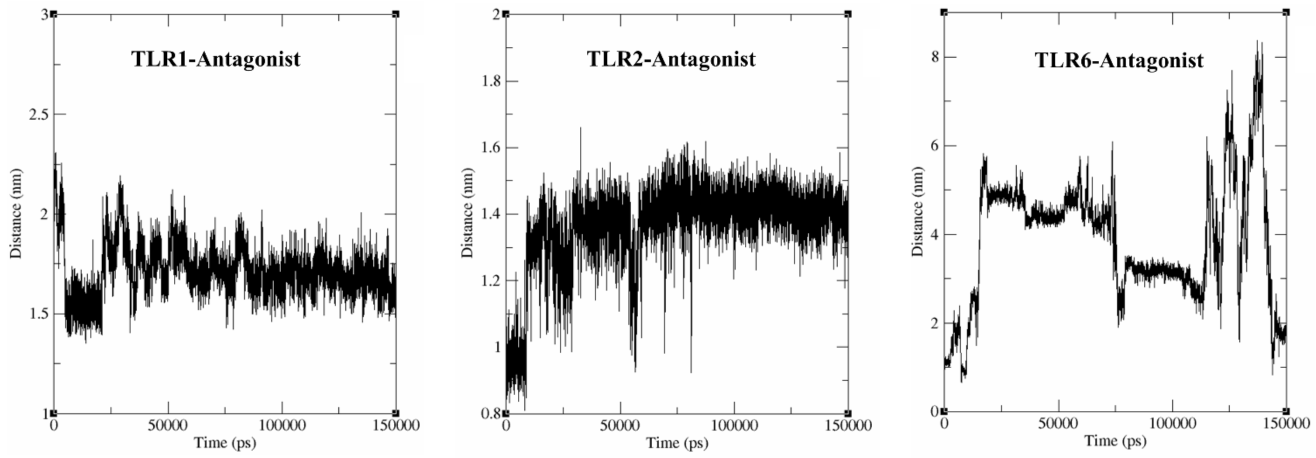

Supplementary Figure S8. The distance analysis between one Oxygen atoms of antagonist and one Carbon alpha of one of the main residues in the main binding site of bovine TLRs during 150 ns MD simulation.

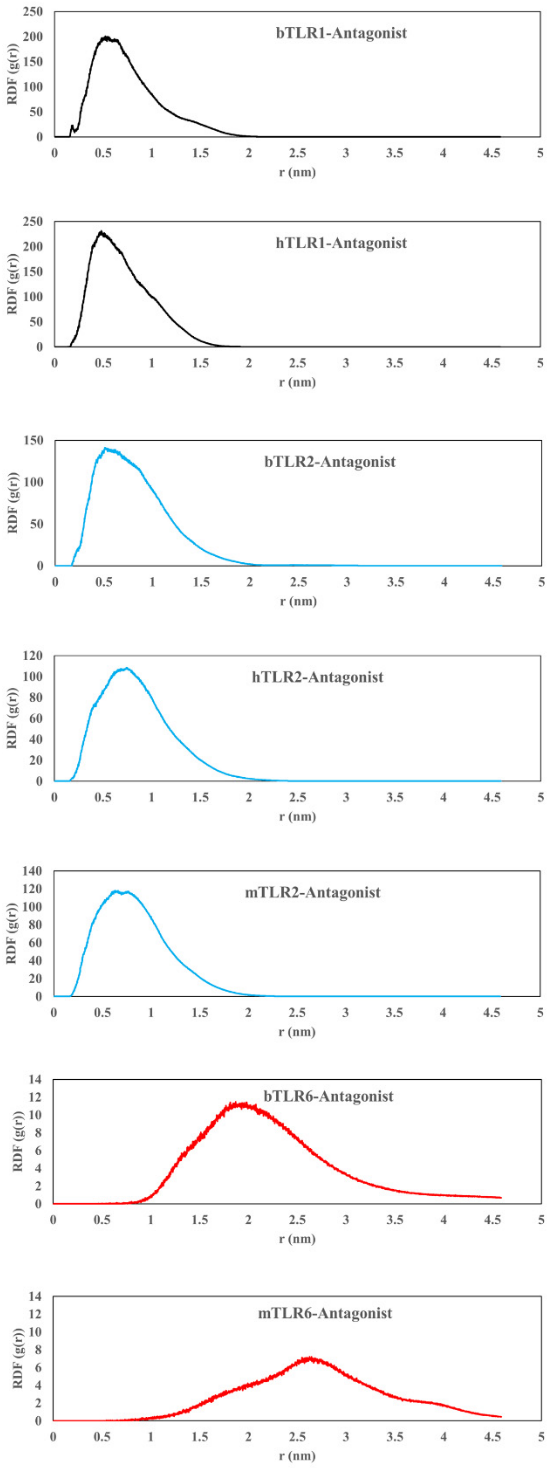

Supplementary Figure S9. RDF analysis for antagonist and the main binding site of TLRs in monomeric forms.

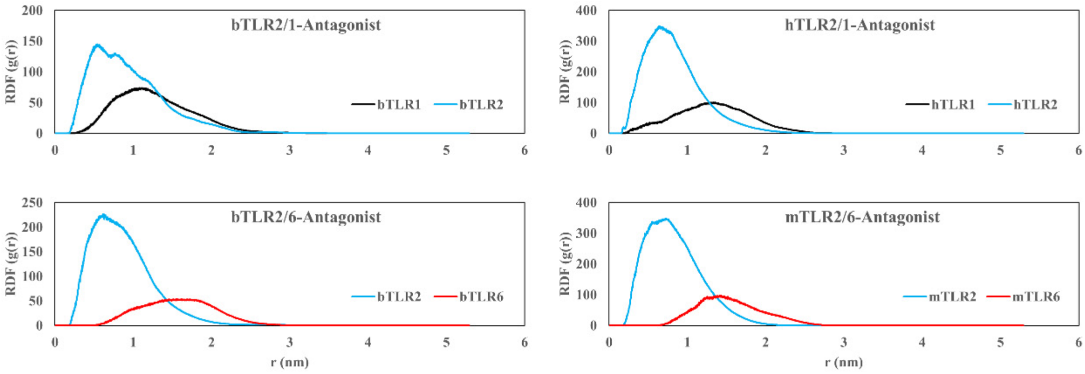

Supplementary Figure S10. RDF analysis for antagonist and the main binding site of TLRs in dimeric forms.

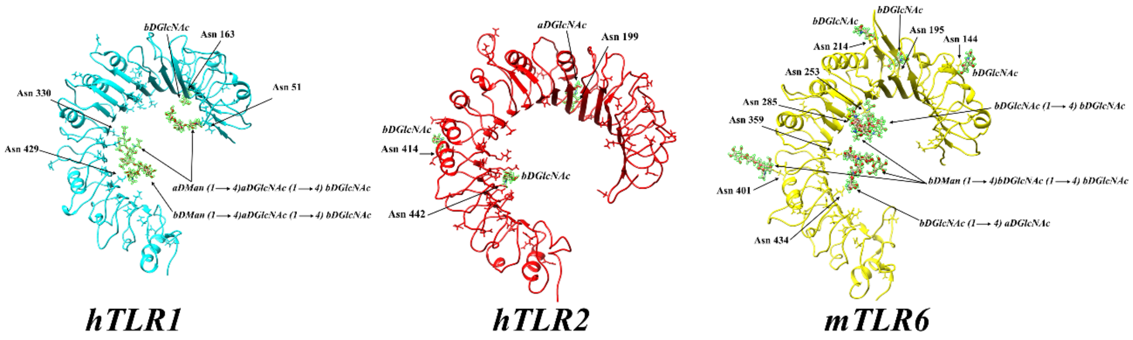

Supplementary Figure S11. Asn residues glycosylated in human and mouse TLRs.

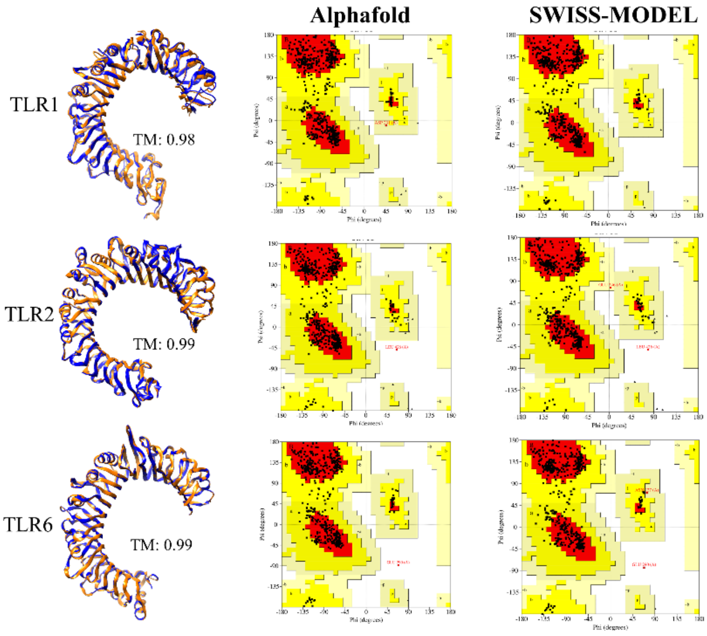

Supplementary Figure S12. Comparison of Alphafold and SWISS-MODEL programs for generating bovine TLRs protein structures. The structural alignment reveals a remarkably high similarity between the protein structures generated by both programs. While the Ramachandran plots exhibit similarities for both Alphafold and SWISS-MODEL, notable distinctions emerge. Interestingly, in SWISS-MODEL structures, the overall quality factor surpasses that of Alphafold. Specifically, for TLR1, the quality factors are 86.7 and 85.5 for SWISS-MODEL and Alphafold, respectively. Similarly, for TLR2, the values are 85.33 (SWISS-MODEL) and 83.17 (Alphafold). Lastly, for TLR6, the quality factors stand at 87.8 and 84.32 for SWISS-MODEL and Alphafold, respectively.

TLR2/1 – PAM3 TLR2/6 – PAM2

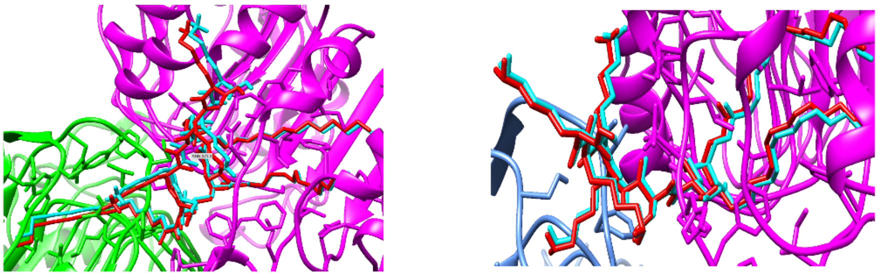

Supplementary Figure S13. Superimposition of extracted PAM3 and PAM2 crystal structures (coordinates extracted from human and mouse TLR2 heterodimers) shown in red, with their docked conformers in cyan, aligned to bovine TLRs.

hTLR2/1-PAM3 ***mTLR2/6-PAM2***

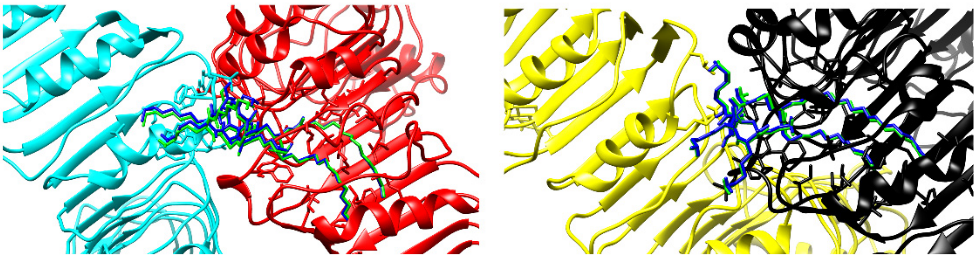

Supplementary Figure S14. Superimposition of PAM3 and PAM2 crystal structure (blue) with its docked conformer (green).

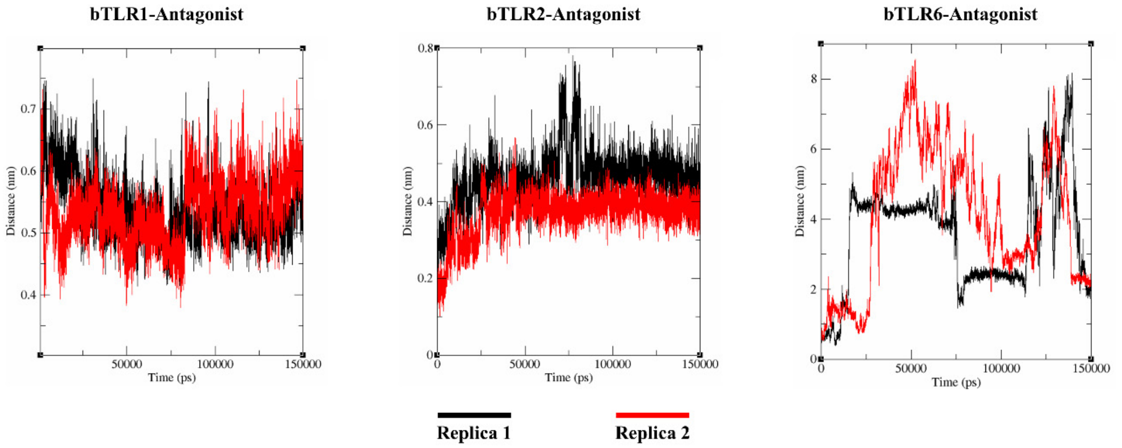

Supplementary Figure S15. Replication analysis for ensuring data reproducibility. The figure illustrates the result of two replicas in our simulation system, conducted to validate the reproducibility of data. The experiments were specifically carried out for bovine TLRs and antagonist systems specifically conducted to the center of mass between TLRs and antagonist. The consistent results across the replica experiments affirm the reliability and reproducibility of the data obtained.

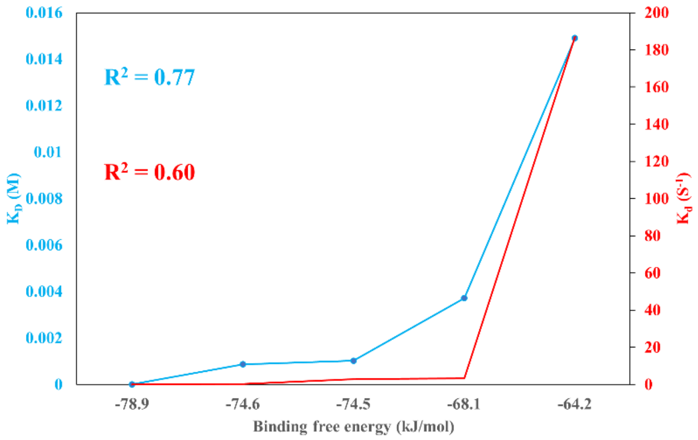

Supplementary Figure S16. The calculated correlation coefficient (R^2) demonstrates the relationship between the binding free energy, determined through MM/PBSA, and the experimental K_D and K_d data. These results affirm the reliability and validity of the MD and MM/PBSA outcomes.

Supplementary Table S1. Ramachandran analysis of TLR structures in three species. This table provides the percentage of residues within favored, allowed, and disallowed regions for TLR structures.

Region	Region colour	*bTLR2*	*hTLR2*	*mTLR2*	*bTLR1*	*hTLR1*	*mTLR1*	*bTLR6*	*hTLR6*	*mTLR6*
Residues in most favoured regions (A, B, L)	Red	82.60%	80.10%	81.30%	79.80%	79.60%	82.40%	77.80%	85.30%	82.90%
Residues in additional allowed regions (a, b, l, p)	Yellow	16.80%	17.50%	17.30%	19.10%	19.40%	17.60%	20%	15%	15.50%
Residues in generously allowed regions (~a, ~b, ~l, ~p)	Pale yellow	0.40%	2.20%	1.40%	1%	1%	0%	1.60%	0.00%	1.70%
Residues in disallowed regions	White	0.20%	0.20%	0%	0.10%	0%	0%	0.60%	0.20%	0%
		99.8 % (allowed)<br>0.2 % (disallowed)	99.8 % (allowed)<br>0.2 % (disallowed)	100 % (allowed)	99.9 % (allowed)<br>0.1% (disallowed)	100 % (allowed)	100 % (allowed)	99.4 % (allowed)<br>0.6 % (disallowed)	99.8 % (allowed)<br>0.2 % (disallowed)	100 % (allowed)

Supplementary Table S2. Binding free energies (Kcal/mol) using MM/PBSA method for bTLR dimers in the last 10 ns of the MD simulation.

	In presence of agonist	In absence of agonist	Initial structure from HADDOCK	Avg.	SD
bTLR2 - bTLR1	448.8	420.8	401.4	423.7	19.5
bTLR2 - bTLR6	339.6	239.9	142.3	240.6	80.5

Supplementary Table S3. HADOCK results for bovine TLR2 dimers.

	HADDOCK score	RMSD	vdW	Electrostatic energy	Desolvation energy	Z-score
**TLR2/1**	-108.3	1.1	-40.8	-320.6	-3.3	-1.5
**TLR2/6**	-137.7	0.6	-64.7	-345.1	-4.1	-1

Supplementary Table S4. The type and number of TLRs residues which involved in the interaction with PAMs.

	Total residue number	Non-polar residues	Polar residues	Polar residues in the pocket
bTLR2 (TLR2/1-PAM3)	17	12	5	4
hTLR2 (TLR2/1-PAM3)	23	19	4	1
bTLR1 (TLR2/1-PAM3)	10	5	5	3
hTLR1 (TLR2/1-PAM3)	10	6	4	2
bTLR2 (TLR2/6-PAM2)	21	13	8	4
mTLR2 (TLR2/6-PAM2)	29	22	7	3
bTLR6 (TLR2/6-PAM2)	6	3	3	0
mTLR6 (TLR2/6-PAM2)	2	1	1	0

Supplementary Table S5. The detilas of docking for ligands and TLRs protein.

	Grid center (xyz)	Grid number (xyz)	Space (Å)
Human TLR2/1	139 * 4.7 * 26	122 * 110 * 66	0.375
Mouse TLR2/6	81.6 * 82.5 * 48.3	98 * 56 * 66	0.375
Bovine TLR2/1	139 * 4.7 * 26	124 * 110 * 66	0.375
Bovine TLR2/6	81.6 * 82.5 * 48.3	98 * 56 * 66	0.375
