# Supplementary material for: Homology Modeling, Molecular Dynamics Simulation, and Prediction of Bovine TLR2 Heterodimerization
Source: Int J Mol Sci. 2024 Jan 25;25(3):1496. doi: 10.3390/ijms25031496 (PMC10855669; doi:10.3390/ijms25031496)
Supplement: Supplementary file 1 [file ijms-25-01496-s001.zip › ERRAT.zip!/mouse-TLR1.pdf]

Program: ERRAT2
File: mouse_TLR1.pdb
Chain#:A
Overall quality factor**: 88.679

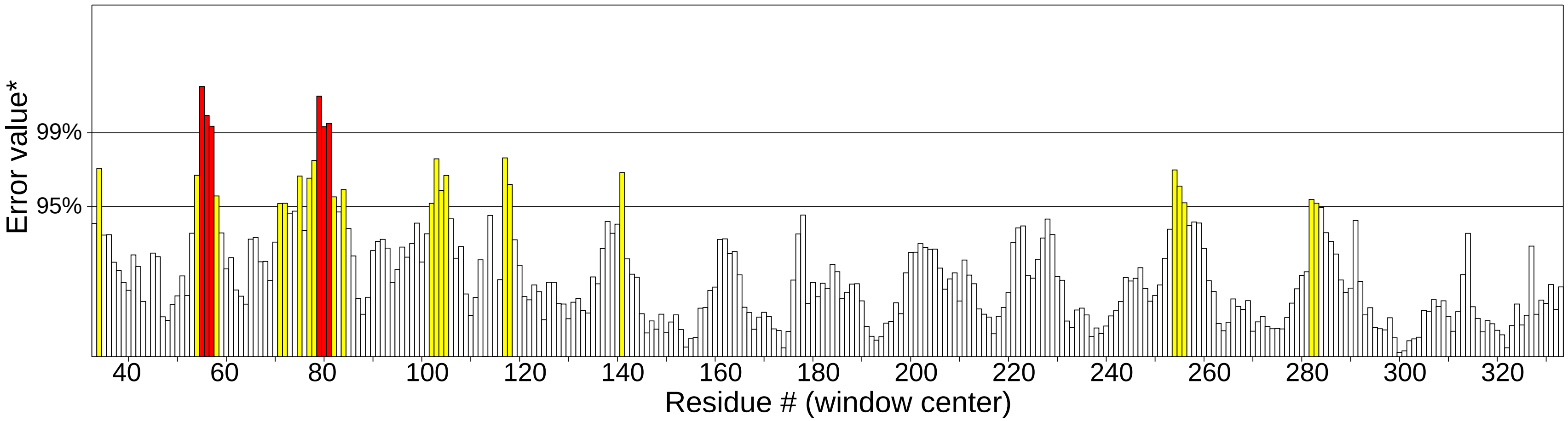


*On the error axis, two lines are drawn to indicate the confidence with which it is possible to reject regions that exceed that error value.

**Expressed as the percentage of the protein for which the calculated error value falls below the 95% rejection limit. Good high resolution structures generally produce values around 95% or higher. For lower resolutions (2.5 to 3A) the average overall quality factor is around 91%.

Program: ERRAT2
File: mouse_TLR1.pdb
Chain#:A
Overall quality factor**: 88.679

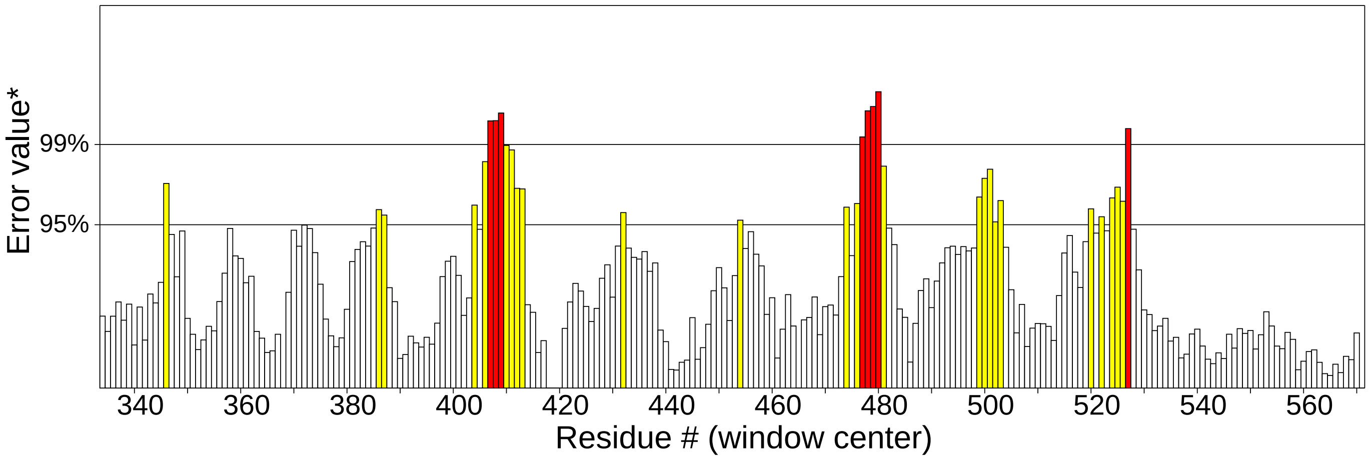


*On the error axis, two lines are drawn to indicate the confidence with which it is possible to reject regions that exceed that error value.

**Expressed as the percentage of the protein for which the calculated error value falls below the 95% rejection limit. Good high resolution structures generally produce values around 95% or higher. For lower resolutions (2.5 to 3A) the average overall quality factor is around 91%.
